# Effectiveness of exercise intervention on fall-related fractures in older adults: a systematic review and meta-analysis of randomized controlled trials

**DOI:** 10.1186/s12877-020-01721-6

**Published:** 2020-09-04

**Authors:** Qiang Wang, Xiaowei Jiang, Yingchao Shen, Ping Yao, Jun Chen, Yuan Zhou, Yunfeng Gu, Zhiyuan Qian, Xi Cao

**Affiliations:** Department of Orthopaedics, Changshu Hospital Affiliated to Nanjing University of Chinese Medicine, No. 6 Huanghe Road, Changshu, 215500 China

**Keywords:** Exercise, Fracture risk, Older adults, Meta-analysis

## Abstract

**Background:**

Exercise intervention can significantly improve physical function and bone strength; however, the effect of exercise on fall-related fractures in older adults remains controversial. This study aimed to assess the effectiveness of exercise intervention on fall-related fractures in older adults by conducting a meta-analysis of randomized controlled trials (RCTs).

**Methods:**

PubMed, EMBASE, and Cochrane databases were systematically searched for RCTs through November 24, 2019 to investigate the effectiveness of exercise intervention on fall-related fractures in older adults. Pooled relative risk (RR) with 95% confidence interval (CI) was calculated using the random-effects model. Sensitivity, subgroup, and publication bias analyses were also conducted.

**Results:**

A total of 7704 older adults and 428 fall-related fracture events from 20 RCTs were selected for the final meta-analysis. The follow-up duration across included trials ranged from 6.0 months to 7.0 years. The pooled RR suggested that exercise intervention was associated with a reduced fall-related fracture risk in older adults (RR: 0.74; 95% CI: 0.59–0.92; *P* = 0.007; *I*^*2*^ = 12.6%). The pooled conclusion was robust and not affected by any individual trial. Subgroup analysis revealed that the significant effect of exercise intervention on fall-related fractures was mainly detected when the study reported results from both male and female subjects, when it did not report the baseline body mass index, when individuals received both home- and center-based interventions, when the follow-up duration was > 1.0 year, and when it was a high-quality study.

**Conclusions:**

Regular exercise intervention could prevent fall-related fractures in older adults. Further large-scale RCTs should be conducted to assess the effectiveness of different exercise programs on fall-related fractures at various sites.

## Background

Falling is the most common cause of fractures in older adults, and fall-related injuries are the leading cause of increased hospitalization and medical costs [1–3]. Fractures occurred in older adults could affect the activities of daily living, even mortality in serious cases [4, 5]. Nearly one-third of the individuals aged ≥65 years fall each year, with 22–45% sustaining injuries and one-tenth sustaining severe injuries such as a fracture or head injury [6–8]. Injurious falls and fractures cause long-lasting functional decline and taking care of such older adults becomes increasingly difficult [9, 10].

Several risk factors for falls and fall-induced injuries have already been demonstrated, which are related to physical inactivity and decreased functional capacity. These factors could be modified by physical activity [11]. However, frequent walking might increase fracture risk in older adults because of increased exposure to fall hazards [12]. The association between exercise intervention and reduced fall risk in older adults has already studied [13–15]. Moreover, numerous systematic review and meta-analyses have already been performed to investigate the role of exercise interventions on the risk of falls, injurious falls, and fractures [16–21]. However, previous studies did not focus on fall-related fractures [16–19, 21], and one study showed a pooled result based on 15 randomized controlled trials (RCTs) [20]. Therefore, the present meta-analysis of RCTs was conducted to assess the effect of exercise intervention on fall-related fractures in older adults.

## Methods

### Data sources, search strategy, and selection criteria

This study was conducted and reported in accordance to the Preferred Reporting Items for Systematic Reviews and Meta-Analysis Statement issued in 2009 [22]. This systematic review and meta-analysis of RCTs investigated the effectiveness of exercise intervention on fall-related fractures in older adults; no restrictions were placed on publication language and status. The electronic databases of PubMed, EMBASE, and Cochrane library were systematically searched for studies through November 24, 2019 using the core search terms “exercise” AND “fracture,” then filtered with “clinical trial” and “Adult: 19–44 years aged >65 years.” The details of search strategy in PubMed are presented in Supplement 1. The reference lists of retrieved studies were reviewed by manual searches to select any additional eligible study.

Two reviewers independently conducted the literature search and study selection. The selection process was based on the participants, intervention, control, outcome, and study design *(PICOS)*. Any disagreement between the two reviewers was resolved by discussion until a consensus. A study was included if it met the following inclusion criteria: (1) study design: RCT; (2) participants: adults aged ≥50.0 years; (3) intervention: exercise program without the use of hormone replacement therapy, glucocorticoids, or bisphosphonates; (4) control: usual care; and (5) outcome: fall-related fracture incidence, irrespective of the site. Studies designed as observational studies, meta-analyses, letters to the editors, and animal studies were excluded.

### Data collection and quality assessment

Two reviewers assessed the abstracted data and study quality following a standardized extraction form, and any disagreement was resolved by an additional author referring to the original article until a consensus was reached. The abstracted items included the first author’s name; publication year; country; sample size; mean age; the percentage of male subjects; body mass index (BMI); the number of fall-related fractures, exercise regimens, controls, supplements; and follow-up duration. The quality of included studies was evaluated using the Jadad scale based on randomization, blinding, allocation concealment, withdrawals and dropouts, and the use of intention-to-treat analysis [23]. Each item was assigned as “yes,” “no,” or “not mentioned”, and the scoring system ranged from 0 to 5. An RCT score of 4 or 5 indicated high quality.

### Statistical analysis

The effectiveness of exercise intervention on fall-related fractures in older adults was assigned as categorical data, and relative risk (RR) with 95% confidence interval (CI) was applied as an effect estimate. Pooled RR with 95% CI was calculated using the random-effects model due to the underlying variations across the included studies [24, 25]. Heterogeneity across included trials was assessed using *I*^*2*^ and Q statistics, and significant heterogeneity was defined as *I*^*2*^ > 50.0% or *P* < 0.10 [26, 27]. Sensitivity analysis was used for assessing the robustness of pooled conclusions [28]. Subgroup analysis for fracture risk was conducted according to the mean age (≥70.0 years or <  70.0 years), sex (female or both), BMI (≥25.0 kg/m^2^ or <  25.0 kg/m^2^), intervention (home-based, center-based, or both), follow-up duration (≥1.0 years or < 1.0 years), and study quality (high or low), and the difference between subgroups was analyzed using *P*-test for interaction [29]. Publication bias was assessed qualitatively using funnel plot and quantitatively using the Egger’s and Begg’s tests [30, 31]. The inspection level was two-sided, and *P* < 0.05 was considered as significant. Statistical analyses were conducted using STATA (version 10.0; Stata Corporation, College Station, TX, USA).

## Results

### Literature search

**Fig. 1:**
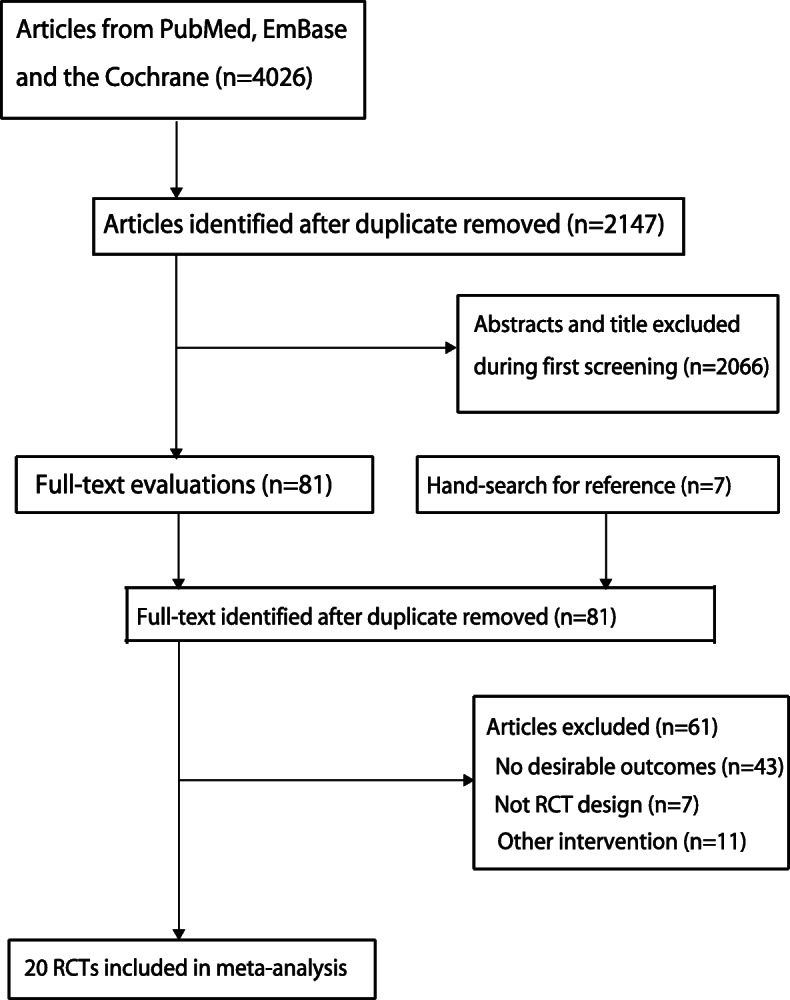
The Preferred Reporting Items for Systematic Reviews and Meta-Analysis Statement (PRISMA) flowchart describing our literature search

A total of 4026 articles were identified from initial electronic searches, of which 1879 were excluded because they were duplicates. An additional 2066 studies were excluded due to the topics being irrelevant. The remaining 81 studies were retrieved for further full-text evaluations; 61 studies were excluded after detailed evaluations. The following were the reasons for exclusion: study did not report fracture incidence (*n* = 43), study did not use RCT design (*n* = 7), and study included other interventions (*n* = 11). A manual search of the reference lists of retrieved studies yielded 7 studies; all studies were contained in electronic searches. Finally, 20 RCTs were selected for the final meta-analysis (Fig. 1) [32–51]. Table 1 summarizes the baseline characteristics of the included studies.

**Table 1:** The baseline characteristics of included studies and participants

Study	Country	Sample size	Age (years)	Percent male (%)	BMI (kg/m^2^)	Number of fractures	Exercise regimen	Control	Supplements	Follow-up duration	Study quality
Preisinger 1996 [32]	Australia	58 (27/31)	60.7 (62.6/59.0)	0.0	NM	7 (5/2)	Postural stability, functional movement, coordination and muscle strength, 3 times per week, for 4 years; home-based	Retained usual lifestyle	No supplements	4.0 years	3
McMurdo 1997 [33]	UK	92 (44/48)	64.5	0.0	NM	2 (0/2)	Weight-bearing exercise, 3 times a week, 10 weeks a year for 2 years; centre-based	Retained usual lifestyle	Daily calcium (1000 mg) supplements	7.0 years	2
Ebrahim 1997 [34]	UK	97 (49/48)	67.2 (66.4/68.1)	0.0	NM	10 (6/4)	Brisk walking for 40 min, 3 times a week, for 2 years; home-based	Received usual care	No supplements	2.0 years	5
Robertson 2001 [35]	New Zealand	240 (121/119)	81.0 (80.8/81.1)	32.5	NM	9 (2/7)	Muscle strengthening and balance exercise, 3 times a week, plus a walking programme, 2 times a week, training for 12 mos and following-up for 18 months; home-based	Usual care	No supplements	1.0 year	5
Chan 2004 [36]	China	108 (54/54)	54.0 (54.4/53.6)	0.0	23.8	3 (1/2)	50 min of Yang Style Tai Chi exercise in each training day, 5 days a week, for 12 months; centre-based	Retained sedentary lifestyle	No supplements	1.0 year	2
Ashburn 2007 [37]	UK	134 (67/67)	72.2 (72.7/71.6)	61.0	NM	8 (2/6)	Daily muscle strengthening, movement, balance training and walking for 6 months; home-based	Received usual care	No supplements	6.0 months	5
Karinkanta 2007 [38]	Finland	144 (108/36)	72.6 (72.8/72.0)	0.0	28.5	4 (2/2)	Resistance training, balance-jumping or combining those exercises together, 3 times a week, for 12 months; centre-based	Asked to maintain Usual physical activity	No supplements	1.0 year	5
Swanenburg 2007 [39]	Switzerland	20 (10/10)	71.2 (71.8/70.7)	0.0	23.3	1 (1/0)	A group programme of muscular strength, co-ordination, balance and endurance training, 3 sessions a week for 3 months, following-up for 12 months; centre-based	Retained routine activities	Daily calcium (500–1000 mg) and Vit D (400–800 IU) supplements	1.0 year	5
Haines 2009 [40]	Australia	53 (19/34)	80.6 (80.9/80.5)	39.6	NM	3 (1/2)	A progressive exercise programme combining lower limb strength and balance exercises, lasting for 6 months; home-based	Retained usual lifestyle	No supplements	6.0 months	5
Zeng 2009 [41]	China	124 (63/61)	50.0–69.0	NA	NM	7 (2/5)	Tai Chi exercise, 3 times a week, training for 2 months and following-up for 2 years; centre-based	Asked to keep usual lifestyle	Daily calcium (600 mg) supplements	2.0 years	3
Bischoff-Ferrari 2010 [42]	Switzerland	173 (87/86)	84.2 (83.4/85.1)	20.8	24.3	22 (7/15)	An additional daily 30-min exercise including balance and strength training, plus functional mobility during acute care, continuing the programme each day after discharge, lasting for 12 months; home-based	Received usual care	No supplements	1.0 year	5
Kemmler 2010 [43]	Germany	227 (115/112)	69.0 (68.9/69.2)	0.0	NM	18 (6/12)	Two times of supervised balance training, strength training and upper body exercises, plus 2 times of home training including strength and flexibility exercises, 4 times a week for 18 months; centre/ home-based	Retained usual lifestyle and exercise habits	Daily calcium (1500 mg) and Vit D (500 IU) supplements	1.5 years	5
Korpelainen 2010 [44]	Finland	160 (84/76)	72.7 (72.7/72.6)	0.0	25.6	38 (16/22)	A weekly supervised balance, leg strength and impact training programme, 6 months each year, followed by a daily home exercise including a similar programme as supervised training, 6 months a year, training for 2 years and following-up for 6 years; centre/home-based	Asked to continue daily routine activities	No supplements	7.0 years	5
Smulders 2010 [45]	Netherlands	92 (47/45)	71.0 (70.5/71.6)	6.3	NM	2 (0/2)	Weight-bearing and walking exercises, and gait correction and fall prevention training programmes, training for 5.5 weeks and following-up for 12 months; centre-based	Usual lifestyle	No supplements	1.0 year	5
Iliffe 2014 [46]	UK	1256 (387/411/458)	73.0	37.6	26.7	9 (3/6)	Centre-based exercise: 1 h of upper body and lower body strength training, plus 2 times of walking per week; home- based exercise: 3 times per week of muscle strengthening and balance exercises plus 2 times of walking; training for 6 months and following-up 1 year; centre/home-based	Retained usual lifestyle	No supplements	2.0 years	5
Gill 2016 [47]	USA	1635 (818/817)	78.9 (78.7/79.1)	32.8	NM	142 (66/76)	The intervention included two center based visits a week and home based activity 3–4 times a week. The physical activity sessions were individualized and progressed toward a goal of 30 min of walking at moderate intensity, 3–5 min of large muscle group flexibility exercises, 10 min of primarily lower extremity strength training by means of ankle weights (two sets of 10 repetitions), and 10 min of balance training; centre/home-based	Health education to continue daily routine activities	No supplements	2.6 years	5
Uusi-Rasi 2017 [48]	Finland	409 (205/204)	74.2 (74.5/74.0)	0.0	NM	15 (8/7)	Static balance, 4 m normal walking speed and five-time chair stand tests, and by the Timed up and go (TUG) test; centre-based	Usual lifestyle	Daily calcium (1098 mg) and Vit D (10.4 μg) supplements	2.0 years	4
Cockayne 2017 [49]	UK	1010 (493/517)	77.9 (78.1/77.7)	39.6	27.7	31 (17/14)	Routine podiatry care as determined by the podiatrist and a falls prevention leaflet in addition to: footwear advice and provision if current footwear was judged to be inappropriate; foot orthoses; and a 30 min a day, three times a week home-based foot and ankle exercise programme supplemented with a DVD and explanatory booklet; centre/home-based	Routine care	No supplements	1.0 year	4
Harris 2019 [50]	UK	1001/297	45.0–75.0/60.0–75.0	36.0/46.0	NM	70 (34/36)	Postal, who received the 12-week PACE-UP walking programme (pedometer, handbook, and PA diary) by post, and nurse- supported, who received the same materials at the first of three practice nurse PA consultations; 12-week PACE-Lift walking programme (pedometer, handbook, PA diary, and feedback on their accelerometry measures) at the first of four practice nurse PA consultations; centre/home-based/centre-based	Routine care	No supplements	3.0 years	4
Liu-Ambrose 2019 [51]	Canada	374 (172/172)	81.6 (81.2/81.9)	33.4	27.0	27 (15/12)	5 strengthening exercises: knee extensor, knee flexor, hip abductor, ankle plantar flexors, and ankle dorsiflexors; 11 balance retraining exercises: knee bends, backward walking, walking and turning around, sideways walking, tandem stands, tandem walking, 1-leg stand, heel walking, toe walking, heel-toe walking backward, and sit to stand; home-based	Usual care	No supplements	1.0 year	5

*BMI body mass index, *NM* not mentioned

### Study characteristics

A total of 7704 older adults and 428 fall-related fracture events from 20 RCTs were included. The publication year of the included studies ranged from 1996 to 2019, and 20–1635 individuals were included in each trial. Overall, 13 studies were conducted in Europe and the remaining seven were conducted in Australia, New Zealand, China, USA, and Canada. A total of nine studies included only females and the remaining 11 included both males and females. Study quality was assessed using the Jadad scale: 13 studies scored 5, three studies scored 4, two studies scored 3, and the remaining two studies scored 2 (Table 2).

**Table 2:** Assessment of risk of bias in included studies

Study	Randomization	Blinding	Allocation concealment	Withdrawals and dropouts	Use of intention-to-treat analysis	Overall
Preisinger 1996 [32]	Yes	Yes	Unclear	Yes	Unclear	3
McMurdo 1997 [33]	Yes	Unclear	Unclear	Unclear	Yes	2
Ebrahim 1997 [34]	Yes	Yes	Yes	Yes	Yes	5
Robertson 2001 [35]	Yes	Yes	Yes	Yes	Yes	5
Chan 2004 [36]	Yes	Unclear	Unclear	Unclear	Yes	2
Ashburn 2007 [37]	Yes	Yes	Yes	Yes	Yes	5
Karinkanta 2007 [38]	Yes	Yes	Yes	Yes	Yes	5
Swanenburg 2007 [39]	Yes	Yes	Yes	Yes	Yes	5
Haines 2009 [40]	Yes	Yes	Yes	Yes	Yes	5
Zeng 2009 [41]	Yes	Unclear	Unclear	Yes	Yes	3
Bischoff-Ferrari 2010 [42]	Yes	Yes	Yes	Yes	Yes	5
Kemmler 2010 [43]	Yes	Yes	Yes	Yes	Yes	5
Korpelainen 2010 [44]	Yes	Yes	Yes	Yes	Yes	5
Smulders 2010 [45]	Yes	Yes	Yes	Yes	Yes	5
Iliffe 2014 [46]	Yes	Yes	Yes	Yes	Yes	5
Gill 2016 [47]	Yes	Yes	Yes	Yes	Yes	5
Uusi-Rasi 2017 [48]	Yes	Yes	Unclear	Yes	Yes	4
Cockayne 2017 [49]	Yes	Yes	Unclear	Yes	Yes	4
Harris 2019 [50]	Yes	Yes	Unclear	Yes	Yes	4
Liu-Ambrose 2019 [51]	Yes	Yes	Yes	Yes	Yes	5

### Meta-analysis

**Fig. 2:**
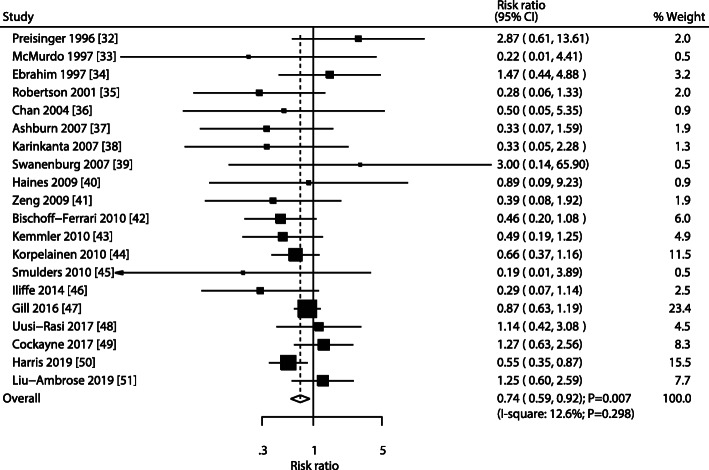
Forest plot for the effect of exercise intervention on the fracture risk in older adults

**Fig. 3:**
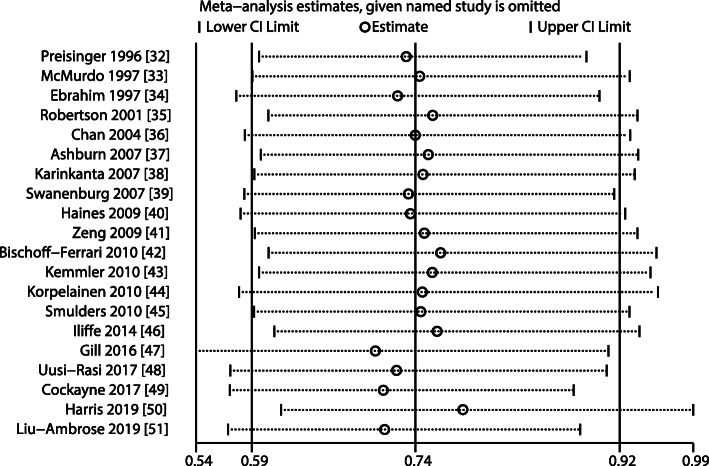
Sensitivity analysis for the fracture risk by sequentially excluding each individual trial

All included studies reported fracture incidence in the exercise and control groups, and the RR across the included trials ranged from 0.19 to 3.00. The pooled RR indicated that exercise intervention significantly reduced fall-related fracture risk (RR: 0.74; 95% CI: 0.59–0.92; *P* = 0.007; Fig. 2). Moreover, unimportant heterogeneity was observed across the included trials (*I*^*2*^ = 12.6%; *P* = 0.298). According to the sensitivity analysis, the pooled conclusion was robust and did not change after sequentially excluding individual trials (Fig. 3).

### Subgroup analysis

Subgroup analysis for fall-related fracture risk between exercise intervention and usual care was also performed. Exercise intervention was associated with reduced fall-related fractures when the study reported both males and females (RR: 0.69; 95% CI: 0.51–0.94; *P* = 0.019), when the study did not report the baseline BMI (RR: 0.72; 95% CI: 0.53–0.96; *P* = 0.024), when individuals received both home- and center-based interventions (RR: 0.71; 95% CI: 0.53–0.96; *P* = 0.025), when the follow-up duration was > 1.0 year (RR: 0.72; 95% CI: 0.55–0.95; *P* = 0.022), and when it was a high-quality study (RR: 0.73; 95% CI: 0.58–0.92; *P* = 0.007). No other significant difference was observed, and the difference between subgroups was not significant, irrespective of stratification by mean age, sex, BMI, intervention, or study quality (Table 3).

**Table 3:** Subgroup analysis for the risk of fracture

Factors	Subgroup	RR and 95%CI	*P* value	Heterogeneity (%)	*P* value for heterogeneity	*P* value between subgroups
Mean age (years)	≥ 70.0	0.78 (0.60–1.01)	0.057	10.6	0.339	0.253
	< 70.0	0.66 (0.42–1.04)	0.075	14.4	0.320	
Gender	Female	0.78 (0.53–1.13)	0.193	0.0	0.440	0.866
	Both	0.69 (0.51–0.94)	0.019	27.4	0.183	
BMI (kg/m^2^)	≥ 25.0	0.81 (0.50–1.33)	0.406	38.0	0.168	0.537
	< 25.0	0.52 (0.24–1.13)	0.100	0.0	0.517	
	Not mentioned	0.72 (0.53–0.96)	0.024	13.5	0.312	
Intervention	Home-based	0.82 (0.45–1.47)	0.500	37.3	0.144	0.829
	Center-based	0.66 (0.34–1.30)	0.233	0.0	0.648	
	Both	0.71 (0.53–0.96)	0.025	34.0	0.181	
Follow-up duration (years)	≤ 1.0	0.75 (0.48–1.15)	0.185	12.4	0.329	0.779
	> 1.0	0.72 (0.55–0.95)	0.022	20.9	0.250	
Study quality	High	0.73 (0.58–0.92)	0.007	14.1	0.292	0.883
	Low	0.75 (0.23–2.46)	0.636	29.4	0.236	

### Publication Bias

**Fig. 4:**
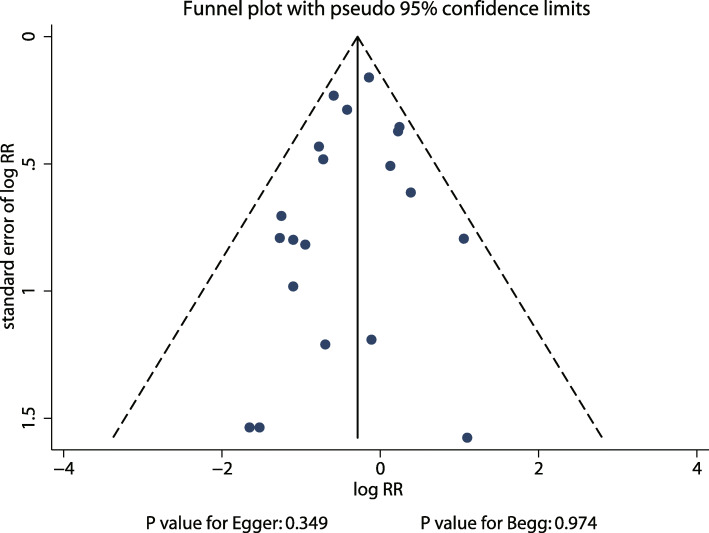
Publication bias test results

Publication bias could not be ruled out by reviewing the funnel plot (Fig. 4). Moreover, the Egger’s (*P* = 0.349) and Begg’s test (*P* = 0.974) results suggested no evidence of publication bias among included trials.

## Discussion

The present study aimed to compare the effectiveness of exercise intervention in reducing fall-related fractures in older adults. This comprehensive quantitative meta-analysis included 7704 older adults and reported 428 fall-related fracture events from 20 RCTs across a broad range of individual characteristics. The findings of this study suggested that exercise intervention reduces fall-related fractures in older adults. It might be anticipated that exercise interventions should be used in older adults for preventing fall-related fractures.

Several systematic reviews and meta-analyses have already described the role of exercise intervention in older adults. Jepsen et al. found that whole-body vibration exercise significantly reduced fall risk [16]. Hill et al. found that exercise intervention had no significant effect on the risk of injurious falls or fractures, although the physical activity, balance, mobility, and muscle strength were significantly improved [17]. Silva et al. suggested that fall risk was significantly decreased when individuals participated in frequent and long-term exercise programs, whereas the fracture risk was not affected by exercise intervention [18]. Kemmler et al. found that the fracture risk was significantly reduced in older adults who participated in exercise interventions [19]. de Souto Berreto et al. pointed out that long-term exercise interventions significantly reduced the incidences of falls, injurious falls, and probable fractures in older adults [21]. The above studies did not focus on fall-related fracture risk in individuals who underwent exercise interventions. A meta-analysis conducted by Zhao et al. included 15 RCTs and found that the risk of fall-related fracture risk, falls, and leg strength were significantly improved in older adults who underwent exercise interventions [20]. However, this study just provided a pooled result between exercise and control for fall-related fracture risk, and whether the treatment effects between exercise and control for fall-related fracture risk differed according to patients’ characteristics was not illustrated [21]. Furthermore, additional five studies have already been published, and the results should have been re-evaluated [47–51]. Although most findings of this study were consistent with a prior meta-analysis [20], the exploratory results of subgroup analysis found significant effects of exercise intervention on the fall-related fracture risk mainly detected when the study reported both male and female subjects, when the study that did not reported baseline BMI, when individuals received both home- and center-based interventions, when the follow-up duration was > 1.0 year, and when it was a high-quality study.

Although most included trials reported similar trends for the protective role of exercise intervention in older adults, several trials reported inconsistent results. Preisinger et al. found that the incidence of vertebral fracture in the exercise group was lower than that in the usual care group, whereas the incidence of fragility fracture in the exercise group was higher than that in the usual care group [32]. These results could be explained by the type of exercise program and the smaller number of events. Ebrahim et al. found that the fracture incidence within 2 years was higher in the exercise group than in the usual care group (6 vs. 4) [34]. The potential reason for this could be the compliance of individuals, risk of injuries, and the intensity of exercise, balancing the potential benefit on fall-related fracture risk. Swanenburg et al. reported that within 12 months, one case fracture was reported in the exercise group and none in the control group; the reason for this was shorter duration and the magnitude of the event occurred was lower than expected [39]. Uusi-Rasi et al. found that fractures related to falls were similar between the exercise and control groups [48]. This result could be explained by the improvement of lower extremity function, mobility, and balance in older adults who underwent exercise interventions [52, 53]. Cockayne et al. found that incidence of fractures in the multifaceted podiatry intervention group was higher than that in the usual care group (17 vs. 14), which may correlate with the type of exercise program [49]. Liu-Ambrose et al. found that individuals who participated in five strengthening exercises and 11 balance retraining exercises may be at an increased fracture risk (RR: 1.93). The potential reason is that older adults with vigorous exercise intervention show low compliance and high incidence of injuries, and low exercise intensity may show limited effects in older adults [54].

Subgroup analyses were conducted, and the results indicated that the beneficial effects of exercise intervention were mainly detected when the study reported both male and female subjects, when the study that did not reported baseline BMI, when individuals received both home- and center-based interventions, when the follow-up duration was > 1.0 year, and when it was a high-quality study. There were several reasons for these results: (1) the incidence of fall-related fracture was lower, and the statistical power in several subgroups was not enough to detect potential differences between the exercise and control groups; (2) the intensity of the exercise program could affect the net effects of exercise on fall-related fractures in older adults; (3) long-term exercise interventions were associated with a reduced incidence of falls in older adults, which could prevent fall-related fractures; and (4) the quality of studies could affect the reliability of individual trials, and this analysis mostly included studies with high quality.

Several limitations of this study should be acknowledged. First, the type and volume of exercise program varies across included trials, which could affect the progression of fall-related fractures. Second, stratified analyses were conducted based on the pooled characteristics of study or patients, and the results stratified by the details of individuals’ characteristics were not available. Third, the analysis of this study was based on published articles, and publication bias was inevitable.

## Conclusion

In conclusion, exercise is an important strategy to decrease fall-related fractures in older adults. Future large-scale RCTs should be conducted to verify the effectiveness of exercise intervention on fall-related fractures at various sites.

## Supplementary information

**Additional file 1.** Supplemental 1 Search Strategy For Pubmed

## Data Availability

All data generated or analyzed during this study are included in this published article and its supplementary information files.

## Abbreviations

RCTs: randomized controlled trials
RR: relative risk
CI: confidence interval
