# Supplementary material for: Effectiveness of exercise intervention on fall-related fractures in older adults: a systematic review and meta-analysis of randomized controlled trials
Source: BMC Geriatr. 2020 Sep 4;20:322. doi: 10.1186/s12877-020-01721-6 (PMC7650290; doi:10.1186/s12877-020-01721-6)
Supplement: Supplementary file 1 — Additional file 1. Supplemental 1 Search Strategy For Pubmed [file 12877_2020_1721_MOESM1_ESM.docx]

**Search strategy in PubMed**

1.  exercis*[Title/Abstract]

2.  training[Title/Abstract]

3.  physical activit*[Title/Abstract]

4. Tai Chi Quan[Title/Abstract] OR Tai Chi exercise[Title/Abstract] OR Tai Chi training[Title/Abstract])

5.  1 OR 2 OR 3 OR 4

6.  fracture[Title/Abstract] OR fractures[Title/Abstract]

7.  fall[Title/Abstract] OR falls[Title/Abstract] OR falling[Title/Abstract]

8.  6 OR 7

9.  5 AND 8

10.  5 AND 8 Filters: Clinical Trial; Adult: 19-44 years; Aged: 65+ years
